# Irradiation Suppresses IFNγ-Mediated PD-L1 and MCL1 Expression in EGFR-Positive Lung Cancer to Augment CD8^+^ T Cells Cytotoxicity

**DOI:** 10.3390/cells10102515

**Published:** 2021-09-23

**Authors:** Chun-I. Wang, Yi-Fang Chang, Zong-Lin Sie, Ai-Sheng Ho, Jung-Shan Chang, Cheng-Liang Peng, Chun-Chia Cheng

**Affiliations:** 1 Radiation Biology Research Center, Institute for Radiological Research, Chang Gung University/Chang Gung Memorial Hospital, Linkou 333, Taiwan; yeewang0330@gmail.com (C.-I.W.); zonlins@gmail.com (Z.-L.S.); 2 Division of Hematology and Oncology, Department of Internal Medicine, Mackay Memorial Hospital, Taipei 104, Taiwan; changyifang@gmail.com; 3 Laboratory of Good Clinical Research Center, Department of Medical Research, Mackay Memorial Hospital, Tamsui District, New Taipei City 251, Taiwan; 4 Department of Medicine, Mackay Medical College, New Taipei City 252, Taiwan; 5 Division of Gastroenterology, Cheng Hsin General Hospital, Taipei 112, Taiwan; aisheng49@gmail.com; 6 Graduate Institute of Medical Sciences, School of Medicine, College of Medicine, Taipei Medical University, Taipei 110, Taiwan; js.chang@tmu.edu.tw; 7 Institute of Nuclear Energy Research, Atomic Energy Council, Taoyuan 325, Taiwan; clpeng@iner.gov.tw

**Keywords:** non-small-cell lung cancer, STAT1, STAT3, PD-L1, MCL1, CD8^+^ T cells, irradiation, radiotherapy

## Abstract

Tumor cells express immune checkpoints to exhaust CD8^+^ T cells. Irradiation damages tumor cells and augments tumor immunotherapy in clinical applications. However, the radiotherapy-mediated molecular mechanism affecting CD8^+^ T cell activity remains elusive. We aimed to uncover the mechanism of radiotherapy augmenting cytotoxic CD8^+^ T cells in non-small-cell lung cancer (NSCLC). EGFR-positive NSCLC cell lines were co-cultured with CD8^+^ T cells from healthy volunteers. Tumor cell viability and apoptosis were consequently measured. IFNγ was identified secreted by CD8^+^ T cells and PBMCs. Therefore, RNAseq was used to screen the IFNγ-mediated gene expression in A549 cells. The irradiation effect to IFNγ-mediated gene expression was investigated using qPCR and western blots. We found that the co-culture of tumor cells stimulated the increase of granzyme B and IFNγ in CD8^+^ T, but A549 exhibited resistance against CD8^+^ T cytotoxicity compared to HCC827. Irradiation inhibited A549 proliferation and enhanced apoptosis, augmenting PBMCs-mediated cytotoxicity against A549. We found that IFNγ simultaneously increased phosphorylation on STAT1 and STAT3 in EGFR-positive lung cancer, resulting in overexpression of PD-L1 (*p* < 0.05). In RNAseq analysis, MCL1 was identified and increased by the IFNγ-STAT3 axis (*p* < 0.05). We demonstrated that irradiation specifically inhibited phosphorylation on STAT1 and STAT3 in IFNγ-treated A549, resulting in reductions of PD-L1 and MCL1 (both *p* < 0.05). Moreover, knockdowns of STAT3 and MCL1 increased the PBMCs-mediated anti-A549 effect. This study demonstrated that A549 expressed MCL1 to resist CD8^+^ T cell-mediated tumor apoptosis. In addition, we found that irradiation suppressed IFNγ-mediated STAT3 phosphorylation and PD-L1 and MCL1 expression, revealing a potential mechanism of radiotherapy augmenting immune surveillance.

## 1. Introduction

Lung cancer is the most common type and the leading cause of cancer-related deaths worldwide [1]. The most common type is non-small cell lung cancer (NSCLC) which comprises 80% of lung cancers [2]. The selection of treatment for NSCLC mainly depends on the diagnostic stage of this disease and the patient’s immune diversity. Basic treatments included surgical resection and radiotherapy (RT) which is often preferred and efficient for patients with early-stages of NSCLC, whereas RT is used at least once in over 60% of lung cancer patients until cure or palliation of this disease [3]. Additionally, sequential chemotherapy with RT or concurrent chemoradiotherapy is also suggested for disease remission in patients with locally advanced stages of lung cancer [4]. Personal medicines targeting specific gene characteristics are currently applied in lung cancers, are resulting in better outcomes for this disease [5]. The main characteristic of NSCLC is EGFR overexpression and mutations, therefore, targeted therapies against EGFR such as tyrosine kinases inhibitors (TKIs) are used in clinical practice [6,7].

Besides, immunotherapy reactivating CD8^+^ T cells such as anti-PD-1 antibodies exhibits tumor therapeutic promise against NSCLC, and results in 20% tumor remission in the patients’ failed treatments of chemotherapies or targeted therapies [8,9,10]. To date, the monoclonal antibodies targeting PD-1 and PD-L1 interaction, such as pembrolizumab and nivolumab for PD-1, and atezolizumab for PD-L1, are approved by FDA for the treatment of metastatic NSCLC [11]. The immune surveillance is responsible for infections and is currently found to potentially eradicate tumors: CD8^+^ T cells recognize tumor cells and secrete granzyme B (*GZMB*) and perforin (*Prf1*) to elicit tumor cell apoptosis and death [12]. Immune checkpoints expressed in tumor cells, including PD-L1 (*CD274*), galectin 9 (*LGALS9*), HVEM (*TNFRSF14*), exhaust CD8^+^ T cells and reduce CD8^+^ T-derived cytotoxicity [13,14,15]. In the tumor microenvironment, IFNγ is secreted by T lymphocytes to reactivate macrophages and CD8^+^ T cells, but it also stimulates PD-L1 overexpression in tumor cells through the JAKs-STAT1 signaling pathway [16]. Meanwhile, the EGFR-STAT3 axis also mediates PD-L1 expression in lung cancer [17]. A case report has revealed a successful tumor therapy by pembrolizumab immunotherapy in TKIs refractory NSCLC [18]. But EGFR-resistance and downstream signaling gene STAT3 activation contributes to cell proliferation, anti-apoptosis, and resistance to CD8^+^ T cells in EGFR-positive lung cancer [19,20].

Irradiation (IR) causes tumor DNA breaks and results in consequent tumor apoptosis. In RT, the accumulation of cytosolic DNA activates the cGAS-STING signaling pathway [21], resulting in type I IFN secretion to potentially reactivate immune surveillance against tumors. According to the current data, it demonstrates that RT enhances the expression of MHC class I [22] and secretion of cytokines such as type I IFN [23], causing an increase in the homing rate of immune cells to the tumor microenvironment [24,25]. Therefore, IR-mediated tumor therapies are considered for improving the anti-tumor efficacy of clinical immunotherapies [26,27,28]. In addition, type I IFN induces PD-L1 expression and PD-L1 expression detection plays a critical role for anti-PD-1 therapies [29,30]. Previous studies have demonstrated that low doses of fractionated IR significantly improve CD8^+^ T cell-mediated tumor remission in combination with anti-PD-1 or anti-PD-L1 therapies [29,31,32]. However, IFNγ from T lymphocytes also activates the JAKs-STATs axis [16,33], which induces the expressions of not only PD-L1 but also cell proliferation and anti-apoptosis genes [34,35]. Since the previous study has indicated that UV light down-regulates IFNγ-mediated STAT1 phosphorylation [36], therefore we proposed a hypothesis that RT may augment CD8^+^ T cell-mediated cytotoxicity through blocking IFNs-mediated activations of JAKs-STATs to suppress immunotherapies-resistant EGFR-positive NSCLC. The aim of this study was to investigate the irradiation effect on IFNγ-mediated STAT1 and STAT3 phosphorylation and genes expression such as PD-L1 and anti-apoptosis genes in EGFR-positive tumor cells to augment CD8^+^ T cells cytotoxicity against NSCLC.

## 2. Materials and Methods

### 2.1. Healthy Volunteers

Blood samples were acquired from healthy volunteers in Mackay Memorial Hospital, Taipei, Taiwan. There were nine volunteers participated in this study. Individual 20 mL of blood was collected, and the peripheral blood mononuclear cells (PBMCs) were harvested in 4 h with the isolation procedures described previously [37]. The study protocol was approved by regulatory authorities and Institutional Review Boards in Mackay Memorial Hospital (20MMHIS018e). Signed and informed written consent were obtained from all participants, and all research was performed in accordance with the relevant guidelines and regulations.

### 2.2. Cell Culture

The lung cancer cell lines were purchased from ATCC and reauthenticated using short tandem repeat analysis in this study (Applied Biosystems, Massachusetts, USA). A549, PC9, and H1650 were cultured in Dulbecco’s Modified Eagle’s Medium (DMEM), and HCC827 was cultured in Roswell Park Memorial Institute (RPMI) 1640 medium supplied with 10% fetal bovine serum (FBS) and 1% penicillin-streptomycin (P/S). All cells were cultured at 37 °C with 5% CO_2_.

### 2.3. Cell Viability

The WST-1 assay (Sigma, Munich, Germany) was used to measure the cell viability according to the manufacturer’s protocol. In brief, 1 × 10^3^ tumor cells were seeded in a 96-well plate, whereas a four-times repeat was necessary for measuring the cell viability in a time-dependent manner. For evaluating the cytotoxic effect of irradiation, A549, HCC827, PC9, and H1650 were exposed to 0, 10, 20 Gy of irradiation and measured after 48 h. For evaluating the cytotoxic effect of PBMCs and CD8^+^ T cells, 1 × 10^5^ PBMCs or 1 × 10^4^ CD8^+^ T cells were co-cultured with 1 × 10^3^ A549, HCC827, PC9, and H1650 cells individually and incubated for 48 h. Moreover, to investigate whether pre-activation of CD8^+^ T cells significantly inhibited the tumor cells, PBMCs were incubated with A549 or HCC827 for 24 h, and CD8^+^ T cells were consequently isolated and co-cultured with the individual pre-incubated A549 or HCC827 for 48 h. RPMI 1640 medium was used to culture the CD8^+^ T cells and PBMCs with tumor cells. The tumor cell viability from co-culture is measured by detected value minus that in PBMCs or CD8^+^ T cells. The same methodology was also used to determine the cytotoxic effect of PBMCs against A549shSTAT1, A549shSTAT3, A549shMCL1, and A549shPDL1 compared to A549shLuc.

### 2.4. Flow Cytometry

The 2 × 10^5^ trypsinized tumor cells were resuspended in 100 μL of RPMI medium and incubated with fluorescent reagents (EGFR-FITC, PDL1-PE, CD133-PE, and CD44-PE antibodies, BioLegend, San Diego, CA, USA) for 30 min at room temperature. Cells were consequently added with 900 μL of PBS buffer containing 1% of FBS and analyzed using an FACSCalibur Attune NxT Flow Cytometer (Invitrogen, Waltham, MA, USA). A commercial kit containing Annexin V-FITC and Propidium Iodide was used (Strong Biotech Corporation, Taiwan) to detect cell apoptosis in tumor cells treated by 0, 10, 20 Gy of irradiation and co-cultured with healthy PBMCs.

### 2.5. Western Blots

The procedure of western blots was as described previously [38]. The specific antibodies against STAT1, pSTAT1(Tyr701), STAT3, pSTAT3(Tyr705), ERK, pERK, and GAPDH were purchased from Cell Signaling (Danvers, MA, USA).

### 2.6. Quantitative Polymerase Chain Reaction (qPCR)

The procedures for mRNA extraction and complementary DNA preparation were same as described previously [38,39]. Quantitative polymerase chain reaction (qPCR) was performed using a SYBR Green system (Applied Biosystems, Foster City, CA, USA) according to the manufacturer’s instruction. The primers are shown in Table 1.

### 2.7. Gene Knockdown

Gene knockdown was conducted using a short-hairpin RNA (shRNA)-expression lentivirus system that contains the specific shRNA (The target sequences are shown in Table 2) in the pLKO.1-puro vector generated by 293T cells. The plasmids were purchased from the National RNAi Core Facility of Academia Sinica, Taipei, Taiwan. The procedure was the same described in our previous study [39]. In brief, 293T cells (70% confluence) cultured in DMEM culture medium were transfected with 4 μg of pLKO.1 vector, 1 μg of the envelope plasmid pVSV-G, and 3.6 μg of the packaging plasmid pCMVΔR8.91. The plasmids were pre-incubated with 6 μL of JetPRIME (Polyplus-transfection, New York, NY, USA) for 20 min at room temperature and consequently added to 293T cells. The cultured medium was substituted with a fresh culture medium after 24 h and further incubated for 48 h. The virus solution was collected and stored at −80 °C. A549 cells cultured in 80% confluence were infected with the prepared lentivirus for 24 h. The cells were then changed with DMEM medium containing 4 µg/mL of puromycin, which was harvested after obtaining stable cells.

### 2.8. Enzyme-Linked Immunosorbent Assay (ELISA) for Measurement of IFNγ

The Human IFNγ PicoKine ELISA (Boster, Pleasanton, CA, USA) was used according to the manufacturer’s protocol to determine IFNγ concentration in the medium of HCC827- and A549-stimulated PBMCs. In brief, 2 × 10^5^ HCC827 and A549 cells were co-cultured with 2 × 10^6^ PBMCs at 37 °C overnight. Then the culture medium was collected and clarified by 2000 rpm centrifugation for 10 min, and each 100 μL of the supernatant medium was added to pre-coated anti-IFNγ antibodies in a 96-well microplate. The consequent steps, including washing, secondary antibodies incubation, and fluorescence detection are completed according to the manufacturer’s instruction.

### 2.9. RNAseq Profiling and Bioinformatics Analysis

RNAseq analysis was performed to investigate IFNγ-induced genes in A549 cell lines by using HiSeq 4000 with paired-end 150 bp sequencing. Genes with >1-fold change (log2) in expression levels in IFNγ-treated A549 compared to parental A549 cells were consequently analyzed using NetworkAnalyst (http://www.networkanalyst.ca/, accessed on 9 December 2020). Pathway activations were selected and matched according to the PANTHER database. Kaplan–Meier plots (http://kmplot.com/analysis/, accessed on 15 December 2020) were used to determine the clinical significance in the overall survival of the patients with lung adenocarcinoma in the dataset (*n* = 865). The expression of selected genes was split by auto selected cutoff.

### 2.10. Isolation of Peripheral Blood Mononuclear Cells (PBMCs) and CD8^+^ T Cells

The procedure for PBMCs and CD8^+^ T cell isolation was the same described previously [37]. In brief, blood samples were collected and analyzed from the individual healthy volunteers (*n* = 9) in Mackay Memorial Hospital, Taipei, Taiwan. 6 mL of buffy coats were isolated from 20 mL of whole blood by an initial 1200 rpm centrifugation for 30 min. The collected 1 mL of buffy coats was then mixed with 7 mL of PBS buffer and loaded onto the 4 mL of ficoll solution and consequently for 2000 rpm gradient centrifugation for 20 min. PBMCs on the interface between the plasma and ficoll media were collected for further analysis. The CD8^+^ T cell isolation kit (Miltenyi Biotec, North Rhine-Westphalia, Germany) was used to isolate CD8^+^ T cells from the PBMCs. The isolation steps were followed based on the manufacturer’s manual.

### 2.11. Chromatin Immunoprecipitation (ChIP)

In brief, 1 × 10^7^ A549 cells were treated with 20 ng/mL of IFNγ for 1.5 h or sequentially treated with 20 ng/mL of IFNγ for 2 h with 20 ng/mL of EGF for 1.5 h. After treatment, A549 cells were fixed by 1% formaldehyde and fragmentized by sonication. A549 cells were resuspended and incubated with control IgG, anti-STAT3, and anti-STAT1 antibodies (Cell Signaling, Danvers, MA, USA) at 4 °C overnight for immunoprecipitation. After incubation, the antibodies were captured by Dynabead-Protein A (Life Technologies, Waltham, MA, USA). The Dynabeads were washed and consequently pulled down by a Sample Magnetic Rack. DNA fragments were eluted by boiling the Dynabeads and concentrated by a FavorPrep GEL/PCR Purification Mini Kit (Favorgen Biotech Corp., Wembley, WA, Australia). The DNA preparation was analyzed by real-time PCR using Fast SYBR Green Master Mix (Applied Biosystem, CA, USA) with primer pairs shown in Table 1. The primer sequences amplify the PDL1 and MCL1 promoter predicted from PROMO (http://alggen.lsi.upc.es/, accessed on 4 May 2021).

### 2.12. Statistical Analysis

GraphPad Prism V8.01 (GraphPad Software, Inc., San Diego, CA, USA) was used to analyze the statistically significant differences, whereas unpaired two-tailed Student’s t-test was used to compare every two group. The tumor cell viability between A549shLuc and A549shRNA treated with same PBMCs (*n* = 10) was calculated based on paired two-tailed Student’s t-test. Moreover, *p* < 0.05 was considered to indicate a statistically significant difference.

## 3. Results

### 3.1. Irradiation and PBMCs Synergistically Inhibited Tumor Cell Viability and Induced Apoptosis in Lung Cancer Cells

**Figure 1:**
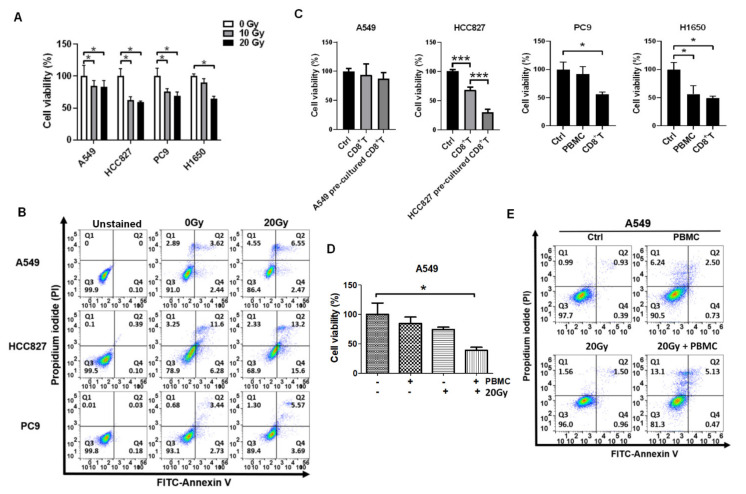
Irradiation improves PBMCs to reduce cell viability and increase apoptosis in EGFR-positive lung cancer cells. (**A**) The cells were exposed with irradiation of 0, 10, 20 Gy. Cell viability was measured after 48 h, and (**B**) the apoptotic cells were detected using flow cytometry after 24 h. (**C**) Cell viability was measured in the EGFR-positive lung cancer cells treated with PBMCs or isolated CD8^+^ T cells for 48 h with a 100-fold number or a 10-fold number, respectively. (**D**) Cell viability was measured in A549 cells in the combined treatment of 20 Gy of irradiation and PBMCs and (**E**) apoptosis was consequently detected by measuring FITC-labeled Annexin V and Propidium iodide (PI). Q1: dead cells; Q2: late apoptosis; Q3: live cells; Q4: early apoptosis. * *p* < 0.05, *** *p* < 0.001.

To investigate whether RT augments immunological surveillance against NSCLC cells, the EGFR-positive tumor cell lines, including A549 (wild-type EGFR; KRAS mutation), HCC827, PC9, and H1650 (EGFR E746-A750 deletion: autophosphorylation), were selected for treatments with irradiation and the isolated healthy PBMCs. Cell viability and apoptosis were then measured. We found that irradiation significantly suppressed cell viability on A549, HCC827, PC9, and H1650 (all *p* < 0.05, Figure 1A). In addition, 20 Gy of irradiation increased late apoptosis rates on A549, HCC827, and PC9 (Figure 1B), and early apoptosis rates on HCC827 and PC9 (Figure 1B). By contrast, CD8^+^ T cells significantly suppressed HCC827, PC9, and H1650 cell viabilities (*p* < 0.05) but did not on A549 (Figure 1C). To increase the CD8^+^ T cells suppressing tumor cells, the healthy PBMCs were incubated with A549 or HCC827 for 24 h, and CD8^+^ T cells were isolated and co-cultured with A549 or HCC827 for 48 h. We noticed that HCC827 pre-incubated CD8^+^ T cells possessed higher cytotoxic efficacy than the parental CD8^+^ T cells to suppress cell viability on HCC827 (*p* < 0.001), but the phenomena was not observed in A549 cells (Figure 1C). Furthermore, the A549 cell line was selected as a CD8^+^ T-resistant model to investigate the synergistic effect of RT on immunological surveillance. We found that combined 20 Gy of irradiation followed by PBMCs treatment significantly led to remarkable inhibition on A549 (*p* < 0.05, Figure 1D), also increased late apoptosis and dead cell rates compared to individual irradiation or PBMCs treatment (Figure 1E).

### 3.2. Reactivation of Healthy CD8^+^ T Cells in Encountering HCC827 and A549 In Vitro

**Figure 2:**
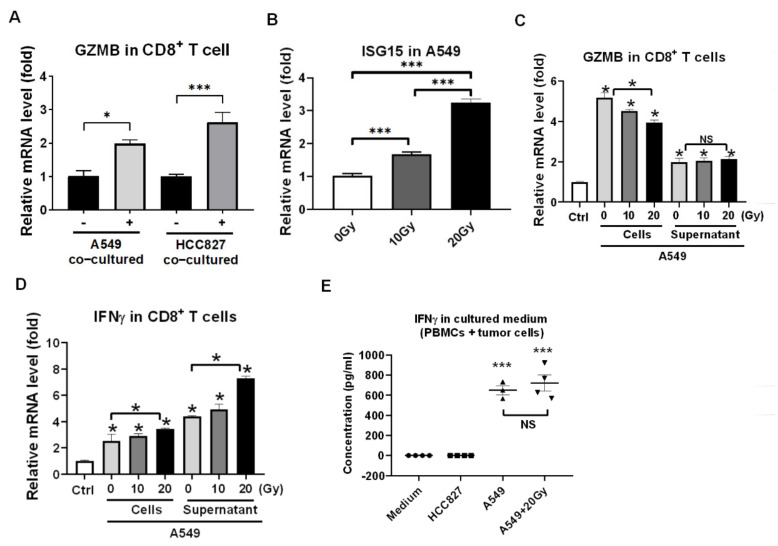
Irradiation improves the reactivation of CD8^+^ T cells in recognizing A549 cells. (**A**) Granzyme B (GZMB) was detected in HCC827- or A549-cocultured CD8^+^ T cells compared to the parental CD8^+^ T cells using qPCR analysis, whereas A549 was resistant to CD8^+^ T cell cytotoxicity but HCC827 was sensitive. (**B**) Interferon-stimulated gene 15 (ISG15) as a marker of irradiation treatment (0, 10, and 20 Gy) in A549 was detected by qPCR analysis. (**C**) GZMB and (**D**) IFNγ were also measured in CD8^+^ T cells cocultured with irradiated A549 or in the collected supernatant medium compared to parental CD8^+^ T cells as control (Ctrl). Irradiated A549 cell lysate significantly increased GZMB levels in CD8^+^ T cells, but irradiated A549 supernatant medium did not. NS: non-significant. Moreover, irradiated A549 cells lysate and collected supernatant medium both increased IFNγ expression in CD8^+^ T cells. (**E**) The secreted IFNγ in the tumor co-cultured PBMCs medium was detected and compared to that in cultured medium using an ELISA analysis. NS, non-significant. * *p* < 0.05, *** *p* < 0.001.

To make sure CD8^+^ T cells were reactivated by tumor cells, the PBMCs were co-incubated with HCC827 and A549 cells for 24 h and the CD8^+^ T cells were isolated, analyzed consequently for the activation markers expression in CD8^+^ T cells using qPCR such as GZMB. The GZMB as the reactivation marker for CD8^+^ T cells and the qPCR results revealed that HCC827 and A549 both activated CD8^+^ T cells (both *p* < 0.05, Figure 2A). Since we demonstrated that RT improved anti-tumor cytotoxicity of PBMCs (Figure 1D,E), the irradiation marker ISG15 was measured and it increased in irradiated A549 in a dose-dependent manner (*p* < 0.001, Figure 2B). The A549 exposed with 0, 10, and 20 Gy of irradiation and the cultured medium was collected 24 h later. The irradiated A549 and the collected medium were incubated with PBMCs for 24 h, and CD8^+^ T cells were isolated consequently for activation markers expression such as GZMB and IFNγ. We found that A549 cells dominantly stimulated the expression of GZMB in the CD8^+^ T cells (*p* < 0.05, Figure 2C) and irradiated A549 supernatant medium dominantly stimulated the expression of IFNγ in the CD8^+^ T cells (*p* < 0.05, Figure 2D). The cultued medium of HCC827, A549, and irradiated A549 was individually collected, and IFNγ was measured using an ELISA assay. We found that A549 and irradiated A549 both stimulated PBMCs to secrete IFNγ in 24 h of incubation, rather than HCC827 (*p* < 0.001, Figure 2E).

### 3.3. IFNγ Dominantly Phosphorylated STAT3 in the Premise of Phosphorylated EGFR

**Figure 3:**
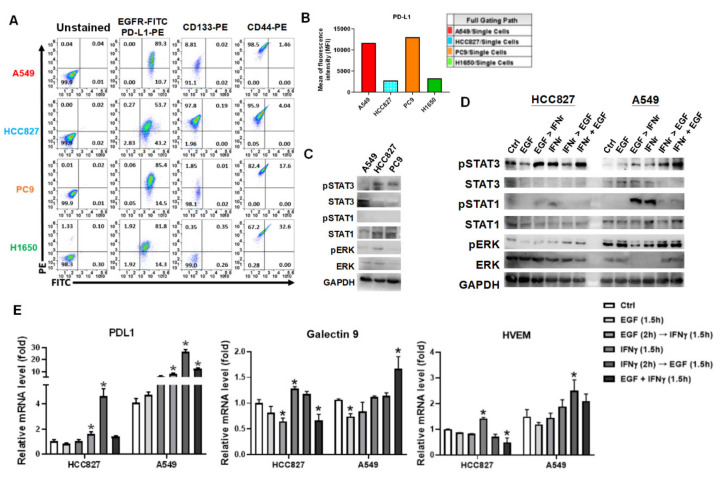
EGF enhances IFNγ-mediated STAT3 phosphorylation and PD-L1 expression. (**A**) Cell surface markers, including EGFR, PD-L1, CD133, and CD44 on NSCLC cell lines were analyzed by flow cytometry. (**B**) EGFR and PD-L1 expression were compared between NSCLC cell lines. (**C**) Protein expression and phosphorylation levels of STAT3, STAT1, ERK, and GAPDH as the internal control were detected in A549, HCC827, and PC9 and (**D**) in HCC827 and A549 treated with indicated treatments by western blots. (**E**) Relative expression levels of immune checkpoints PDL1 (*CD274*), Galectin 9 (*LGALS9*), and HVEM (*TNFRSF14*) were detected by qPCR in HCC827 and A549 treated with indicated treatments. IFNγ and EGF: 20 ng/mL. * *p* < 0.05.

IFNγ is a cytokine secreted by T lymphocytes for further stimulation of B cells and macrophages. IFNγ is considered an activation marker of CD8^+^ T cells. The increase of GZMB and IFNγ in CD8^+^ T cells indicated that the CD8^+^ T cells were reactivated after A549 co-culture, but we found A549 remained resistant to CD8^+^ T cells. The IFNγ-mediated downstream signaling pathways were investigated furthermore, such as STAT1 and STAT3 phosphorylation and PD-L1 expression in A549 cells. A549 (EGFR wild-type) and HCC827, PC9, H1650 (EGFR E746-A750 deletion with auto-phosphorylation) highly expressed EGFR and PD-L1 (Figure 3A). In addition, the four cell lines were CD44-positive, and A549 and HCC827 expressed CD133 higher than PC9 and H1650 (Figure 3A). We noticed that A549 and PC9 expressed EGFR and PD-L1 higher than HCC827 and H1650 (Figure 3B). A549, HCC827, and PC9 were selected and compared for phosphorylations on STAT1 and STAT3. We found intrinsic STAT3 phosphorylation in HCC827 and PC9 cells but no STAT1 phosphorylation was detected in the selected cell lines (Figure 3C). Further investigation revealed that IFNγ increased phosphorylation on STAT1 and STAT3 in HCC827 and A549 (Figure 3D). Interestingly, consequent and synergic EGF treatment highly elicited IFNγ-mediated STAT3 phosphorylation with simultaneous inhibition of STAT1 phosphorylation (Figure 3D). No effect of EGF was found on HCC827 due to the fact that HCC827 was an EGFR autophosphorylated cell line. The immune checkpoints, including PD-L1 (*CD274*), galectin 9 (*LGALS9*), and HVEM (*TNFRSF14*) were measured using qPCR in the arranged treatments. We demonstrated that IFNγ significantly increased PD-L1 (*p* < 0.05) but not Galectin 9 and HVEM in A549 cells (Figure 3E). By contrast, IFNγ increased PD-L1, Galectin 9, and HVEM expression in HCC827 cells (all *p* < 0.05, Figure 3E). Meanwhile, the consequent and synergic EGF treatment increased IFNγ-mediated PD-L1 expression in HCC827 and A549 cells (both *p* < 0.05, Figure 3E). Particularly, EGF suppressed IFNγ-mediated Galectin 9 and HVEM expression in HCC827, but which stimulated Galectin 9 and HVEM expression in A549 (Figure 3E). The different expression of the immune ckeckpoints between HCC827, an EGFR mutant with EGFR autophosphorylation, and A549, an EGFR wild-type with KRAS mutation, warrants further investigated.

### 3.4. IFNγ Increased MCL1 Expression in the Premise of Phosphorylated EGFR

**Figure 4:**
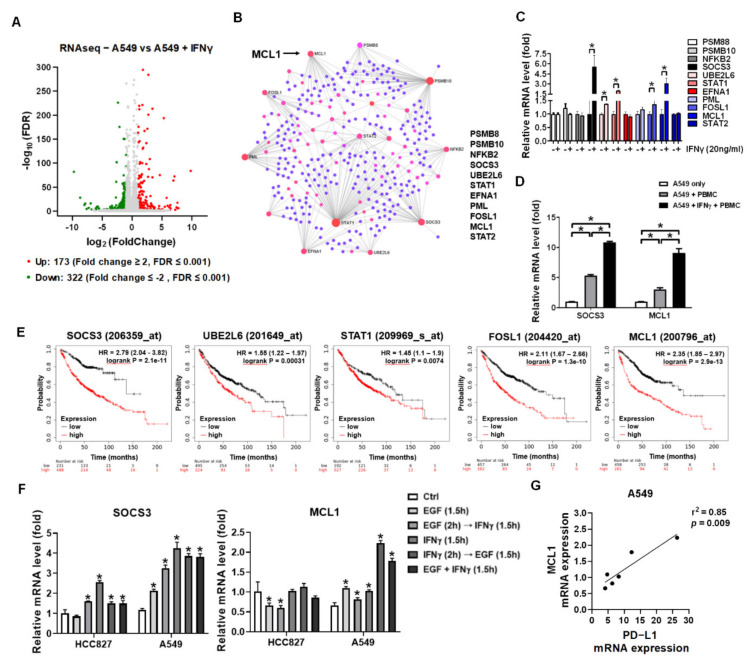
RNAseq reveals IFNγ-mediated gene expression in A549 cells. (**A**) Volcano plot of log 2 (fold change)-log 10 (false discovery rate (FDR)) test was used to distinguish from the differentially expressed mRNAs between IFNγ-treated A549 cells and parental cells (Table S1). Upregulated and downregulated genes in IFNγ-treated A549 cells were labeled as red and green dots, respectively, based on log2 fold changes >1 and <−1 with false discovery rate (FDR) ≤ 0.001. (**B**) The 173 upregulated genes were analyzed and NetworkAnalyst revealed 11 relevant gene candidates, including PSM88, PSMB10, NFKB2, SOCS3, UBE2L6, STAT1, EFNA1, PML, FOSL1, MCL1, and STAT2. (**C**) Relative expressions of the 11 gene candidates in IFNγ-treated A549 cells were validated by qPCR analysis. (**D**) Relative expressions of SOCS3 and MCL1 that associating with apoptosis were detected by qPCR in A549 and IFNγ-pretreated A549 (20 ng/mL of IFNγ for 2h) after cocultured with a 10-fold number of PBMCs for 24h. (**E**) For the genes with significant change, including SOCS3, UBE2L6, STAT1, FOSL1, and MCL1, Kaplan-Meier plots (http://kmplot.com/analysis/, accessed on 15 December 2020) were used to determine the clinical significance in patients with lung adenocarcinoma (*n* = 865). (**F**) Relative expression levels of SOCS3 and MCL1 in HCC827 and A549 with indicated treatments were detected by qPCR. IFNγ and EGF: 20 ng/mL. (**G**) The MCL1 levels in the IFNγ with EGF treatments were correlatively analyzed to the PD-L1 levels from Figure 3E. * *p* < 0.05.

Besides PD-L1 overexpression in A549 to exhaust CD8^+^ T cells, we further investigated the mechanism eliciting resistance against CD8^+^ T cell cytotoxicity in A549 cells. RNAseq was used to investigate the differential gene expression derived by IFNγ treatment. The differential genes >2 and <−2 fold changes with *p*-value < 0.001 were selected and analyzed (Figure 4A and Table S1). NetworkAnalst revealed the driver gene from the IFNγ-increased differential genes, including PSMB8, PSMB10, NFKB2, SOCS3, UBE2L6, STAT1, EFNA1, PML, FOSL1, MCL1, and STAT2 (Figure 4B). After validation using qPCR, SOCS3, UBE2L6, STAT1, FOSL1, MCL1 were significantly increased in IFNγ-treated A549 (*p* < 0.05, Figure 4C). The five genes were associated with survival probability in patients with lung cancer using Kaplan-Meier plotter analysis, which demonstrated that an increase of each gene was associated with a poor survival rate (Figure 4E). SOCS3 and MCL1 were particularly investigated since literature has indicated that they were associated with apoptosis [40] and anti-apoptosis function [41], respectively. PBMCs co-culture significantly increased SOCS3 and MCL1 expression in A549 cells (*p* < 0.05, Figure 4D). Particularly PBMCs elicited higher SOCS3 and MCL1 expression in IFNγ-pretreated A549 cells (*p* < 0.05, Figure 4D). We also found that individual EGF and IFNγ increased SOCS3 expression in A549 (*p* < 0.05, Figure 4F) and consequent and synergic EGF with IFNγ increased MCL1 expression in A549 (*p* < 0.05, Figure 4F). The MCL1 expression was positively correlated with PD-L1 expression in the arranged treatments (r^2^ = 0.85, *p* = 0.009, Figure 4G), but SOCS3 was not (r^2^ = 0.31, *p* = 0.246, data not shown).

### 3.5. Irradiation Specifically Blocked IFNγ-Mediated Phosphorylations on STAT1 and STAT3 in A549 Cells

**Figure 5:**
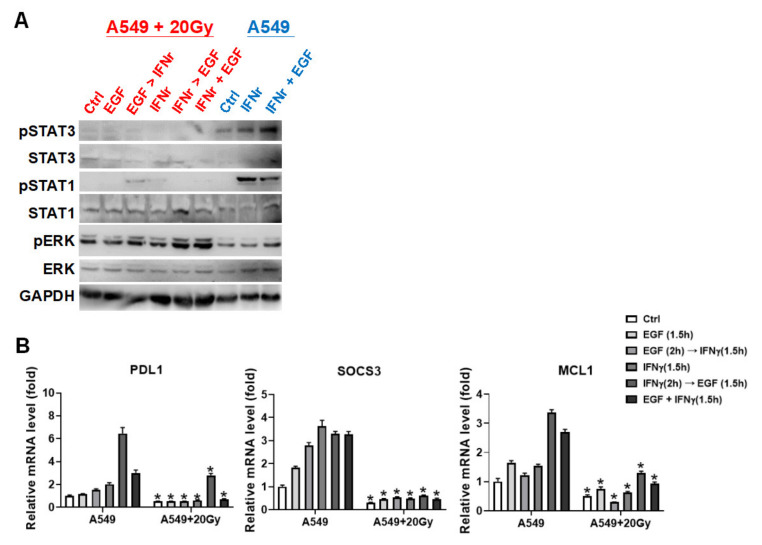
Irradiation specifically suppressed phosphorylation on STAT1 and STAT3. (**A**) Protein expression and phosphorylation levels of STAT3, STAT1, and ERK, and GAPDH as an internal control were detected in IFNγ- with EGF-treated A549 after 24 h exposed by 20 Gy of irradiation. (**B**) Relative expression levels of PDL1, SOCS3, and MCL1 were also detected in A549 treated with the indicated combination of IFNγ and EGF (20 ng/mL) after irradiation exposure. Statistical analysis was calculated between A549 and irradiated A549 with same treatments. * *p* < 0.05.

The IFNγ-mediated phosphorylations on STAT1 and STAT3, and gene expressions on PD-L1, SOCS3, and MCL1 were detected in irradiation-treated A549 cells. The results indicated that 20 Gy of irradiation specifically inhibited IFNγ-mediated STAT1 phosphorylation and synergic effect of EGF with IFNγ on STAT3 phosphorylation (Figure 5A). PD-L1, SOCS3, and MCL1 expressions were consequently reduced in 20 Gy of irradiation treatment compared to the corresponding arranged treatments (all *p* < 0.05, Figure 5B).

### 3.6. STAT3 Dominantly Determined IFNγ-Mediated Gene Expression and Knockdown of STAT3-Mediated MCL1 Augmented PBMCs against A549 Cells

**Figure 6:**
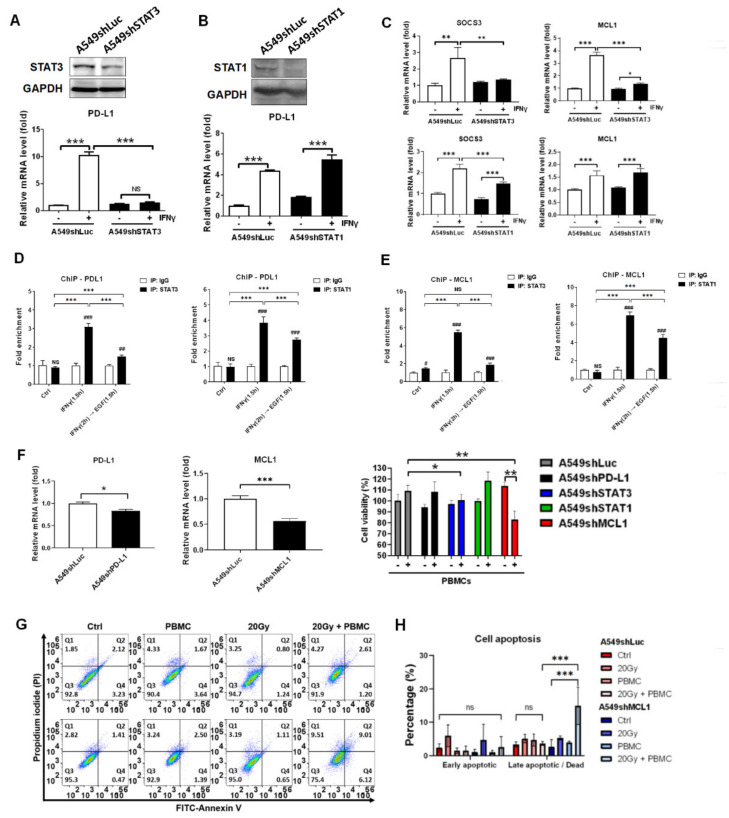
STAT3 determines MCL1 expression and knockdown of MCL1 enhances cytotoxicity of irradiation combined with PBMCs against A549 cells. (**A**) STAT3 and (**B**) STAT1 were knock downed (A549shSTAT3 and A549shSTAT1) and detected by western blots. Relative expression levels of PD-L1, and (**C**) apoptosis-associated gene SOCS3, and MCL1 were detected in IFNγ-treated A549shSTAT3 and A549shSTAT1 compared to A549shLuc by qPCR. (**D**,**E**) Recruitment of STAT3 and STAT1 onto the promoter regions of PDL1 and MCL1 was analyzed in A549 cells treated with IFNγ and followed EGF by ChIP-qPCR. (**F**) Cell viability of A549 cells with specific gene knockdown (PDL1, STAT3, STAT1, and MCL1) was measured after cocultured with a 100-fold number of PBMCs for 48 h incubation (*n* = 9). (**G**,**H**) Cell apoptosis in A549shMCL1 treated with irradiation and PBMCs for 24 h was measured and compared to A549shLuc using flow cytometry. The cells were cocultured with a 20-fold number of PBMCs for 6 h after irradiation exposure. Q1: dead cells; Q2: late apoptosis; Q3: live cells; Q4: early apoptosis. * *p* < 0.05, ** *p* < 0.01, *** *p* < 0.001.

To ensure that irradiation-inhibited phosphorylation on STAT1 and STAT3 augmented PBMCs-mediated anti-tumor effect, STAT1 and STAT3 were knocked down and PD-L1, SOCS3, and MCL1 expressions were investigated. We found that STAT3 knockdown significantly reduced IFNγ-mediated PD-L1 expression (*p* < 0.001, Figure 6A). However, STAT1 knockdown did not affect IFNγ-mediated PD-L1 expression (Figure 6B). We also demonstrated that IFNγ-mediated SOCS3 and MCL1 expressions were decreased in A549shSTAT3 cells (*p* < 0.01 for SOCS3; *p* < 0.001 for MCL1) but not in A549shSTAT1 cells as compared to A549shLuc cells (*p* < 0.001 for SOCS3; non-significant for MCL1, Figure 6C). To clarify the potential regulatory mechanism, we used ChIP to investigate whether STAT3 and STAT1 directly bind to the promoters of individual PD-L1 and MCL1. The results revealed that STAT3 and STAT1 both were able to bind to the promoters of PD-L1 (*p* < 0.001, Figure 6D) and MCL1 (*p* < 0.001 but non-significant in IFNγ + EGF group with anti-STAT3 immunoprecipitation, Figure 6E) in IFNγ and IFNγ with EGF treatments.

Furthermore, PD-L1 and MCL1 were knocked down in A549 cells (Figure 6F). The four stable A549 knockdown cell lines, including A549shPD-L1, A549shSTAT3, A549shSTAT1, and A549shMCL1 were further investigated and compared to A549shLuc after co-cultured with healthy PBMCs. We found that cell viability was decreased in A549shSTAT3 (*p* < 0.05) and A549shMCL1 (*p* < 0.01) compared to A549shLuc in PBMCs treatment (Figure 6F). Knockdown of PD-L1 and STAT1 did not improve PBMCs cytotoxicity against A549 (Figure 6F). To address and validate that MCL1 elicited resistance to the cytotoxicity of PBMCs, apoptosis was measured in A549shMCL1 with irradiation and PBMCs treatments. The results demonstrated that knockdown of MCL1 increased A549 cell death rate in 20 Gy with PBMCs treatment compared to control A549shLuc cells (*p* < 0.001, Figure 6G,H).

## 4. Discussion

In this study, we used healthy PBMCs to investigate the mechanism of RT augmentation to immune surveillance. To our knowledge, CD8^+^ T cells are exhausted in patients with tumors. Exhausted CD8^+^ T cells are characterized by loss of cytotoxic functions with increased expression of multiple inhibitory receptors such as PD-1 and decreased secretion of effector cytokines such as IFNγ [42]. Current immunotherapies are mainly designed to reactivate the immune system in tumor patients. Reactivation of the exhausted CD8^+^ T cells with inhibitory receptor blockade promotes immunity and survival outcome in tumor patients [43]. Besides, CD8^+^ T cells can also be activated by inserting a specific TCR recognizing tumor antigen called chimeric antigen receptor T-cell therapy (CAR-T) [44]. Once activated in CD8^+^ T cells, IFNγ is secreted, and the function of which is to bind and activate macrophages to induce M1 differentiation [45,46]. However, IFNγ also induced JAKs-STATs signaling pathways in tumor cells [16]. We clarified the potential IFNγ-mediated mechanism resisting the cytotoxicity of CD8^+^ T cells through expressing MCL1 in EGFR-positive NSCLC in this study.

We have previously demonstrated that CD16 (FcγRIIIA) and CD122 (IL2/IL15Rβ) are correlated in the healthy CD8^+^ T cells [37]. To our knowledge, CD16 on CD8^+^ T cells acts as an activation site exerting antibody-dependent cellular cytotoxicity (ADCC) function, the levels of which are decreased in exhausted CD8^+^ T cells treated by nicotine treatment and in smokers [37]. CD16^+^ CD8^+^ T cells exhibit natural killer (NK)-like and are terminally differential memory effector T phenotype with high levels of granzyme B and perforin [47,48], indicating this small group of effector CD8^+^ T cells may exhibit anti-tumor potential since granzyme B and perforin causes tumor apoptosis. The unconventional NK-like [KIR/NKG2A(+)Eomes(+)] CD8^+^ T cells with TDEM phenotype [CD45RA(+)CCR7(-)] have innate features and able to secrete IFNγ rapidly following stimulations compared to conventional memory [KIR/NKG2A(-)Eomes(+)] CD8^+^ T cells [49]. Moreover, CD16 is frequently increased in the unconventional NK-like CD8^+^ T cells [50]. To our knowledge, Eomes determines the differentiation of CD8^+^ T cells into effector and memory phases and regulates CD122 expression [51]. CD122 is the receptor of IL2 and IL15 contributing to T cell proliferation and granzyme B expression through activating JAKs-STAT5. Literature has indicated that IL15 can substantially increase CD16 expression in CD8^+^ T cells [50]. In addition, IL2 is used in clinical applications to suppress tumors. Based on the evidence, CD16^+^ CD122^+^ CD8^+^ T cells may reflect the reactivation of CD8^+^ T cells as a biomarker of cancer.

EGFR overexpressed in the selected cell lines, including A549, HCC827, PC9, and H1650 (Figure 3A). The EGFR autophosphorylation has been reported in HCC827 and PC9, these two cell lines carrying EGFR exon 19 deletion, which may lead to high phosphorylation of STAT3 (Figure 3C). Although we demonstrated that irradiation significantly suppressed the cell viability in the EGFR-positive lung cancer cell lines, a previous literature has demonstrated that repeated irradiation exposure induces EGFR expression and activation [52]. The induced expression of mutated EGFR with autophosphorylation consequently causes activations of downstream signaling pathways, resulting in highly cell proliferation and survival against irradiation therapies. Therefore, EGFR tyrosine kinase inhibitors considered be effective to EGFR mutations are potential as radiosensitizers [53,54]. Besides, other factors activated by EGFR such as the EGFR downstream STAT3 has also been demonstrated contributing to radioresistance as a therapeutic target reverses radioresistance in lung cancer [55]. Since we found that irradiation specifically suppressed STAT1 and STAT3 phosphorylation in IFNγ-treated A549 cells in this study, we speculated that STAT3 caused radioresistance was mediated by overexpression and activation of mutated EGFR in lung cancer after survived in RT treatment.

Since the characteristic of autophosphrylation of EGFR exon 19 deletion, EGF did not affect STAT3 phosphorylation but IFNγ caused highly STAT3 phosphorylation in HCC827. The same phenomena were observed in the sequential treatment of EGF and IFNγ in A549 (Figure 3D). It also validated and revealed that STAT3 phosphorylation suppressed STAT1 phosphorylation, indicating the possibility of lower levels of PD-L1 in HCC827 compared to that in A549. We speculated that low levels of STAT1 and pSTAT1 in HCC827 resulted in susceptibility to the cytotoxicity of CD8^+^ T cells since we demonstrated that STAT1 bound to the promoter of PD-L1 and MCL1. Compared to HCC827, there was an instant increase of MCL1 in the sequential IFNγ with EGF-treated A549 (Figure 4F). Meanwhile, the JAKs-STATs signaling axis also regulated MHCI expression [56,57]. We found that IFNγ specifically increased non-classical MHCI HLA-E molecules which is the ligand of CD94/NKG2 receptors expressed in NK cells and a subset of CD8^+^ T cells [58]. Therefore, IFNγ-pretreated A549 exhibited a higher immunogenic capacity to immunity. We found that IFNγ-pretreated A549 also expressed highly MCL1 expression responding to PBMCs (Figure 4D). This study provides evidence suggesting that the IFNγ-mediated JAKs-STATs axis plays a double-edged sword in NSCLC stimulating reactivation of CD8^+^ T cells but increasing anti-apoptosis MCL1 expression in A549.

Evidence shows that RT enhances the reactivation of CD8^+^ T cells against NSCLC through specifically inhibiting phosphorylation of STAT1 and STAT3 and reducing the IFNγ-mediated anti-apoptosis MCL1 expression. IR is considered to break DNA and lead to consequent tumor apoptosis as a reliable tumor therapy. Although previous studies have indicated that tumor cells under IR-mediated stress trends to stimulate STAT3 activation for spontaneous defense to DNA damage and cellular apoptosis [59]. With sufficient IR strength causing irreversible progress such as 20 Gy in this study, cellular cytosolic DNA activates the cGAS-STING signaling pathway and results in type 1 IFN-mediated ISG15 expression. ISG15, therefore, secreted by IR-treated tumors plays as a cytokine that may activate CD8^+^ T cells through binding to the LFA1 receptor, resulting in IFNγ expression in CD8^+^ T cells [60]. We found that HCC827 did not induce secretion of IFNγ in the co-co-cultured PBMCs (Figure 2E), that may be loss of cGAS-STING activation in the apoptotic HCC827 demonstrated previously in the cisplatin-treated HCC827 [61]. The activation of cGAS-STING is considered to secrete type I interferons [21] which induces IFNγ expression in immune cells [62]. Interestingly, this study demonstrated that IR suppressed phosphorylation on STAT1 and STAT3 specifically. We speculated that SHP1/2 may be activated by IR [63,64], which dephosphorylated STAT1 and STAT3. The detailed mechanism for irradiation suppressing IFNγ-mediated STAT1 and STAT3 phosphorylation warranted further investigation.

## 5. Conclusions

**Figure 7:**
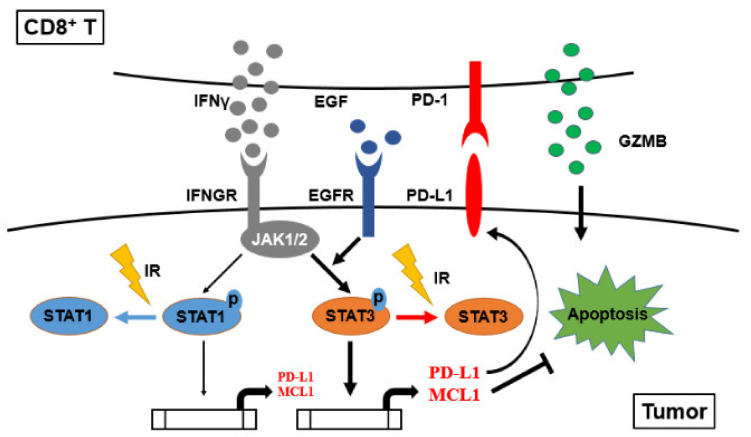
The schematic diagram illustrates the potential mechanism of irradiation augmenting CD8^+^ T cells against NSCLC. IFNγ secreted by activated CD8^+^ T cells stimulates PD-L1 and MCL1 expression through phosphorylating STAT1 and STAT3 in EGFR-positive lung cancer. Irradiation (IR) was demonstrated to inhibit IFNγ-mediated phosphorylation on STAT1 and STAT3, that reduced MCL1-mediated anti-apoptosis and enhanced CD8^+^ T cell-mediated cell apoptosis against CD8^+^ T cells-resistant A549 cells.

Healthy CD8^+^ T cells were reactivated in co-cultured with HCC827 and A549, resulting in overexpression of granzyme B (GZMB) and IFNγ. Granzyme B was considered a hydrolase enzyme leading to tumor cell apoptosis but IFNγ induced STAT3-mediated PD-L1 and MCL1 expression in EGFR-positive lung cancer cells. We demonstrated that IR elicited A549 cell apoptosis and augmented PBMCs-mediated tumor cell death through prohibiting IFNγ-mediated phosphorylation of STAT1 and STAT3 and gene expression of PD-L1 and MCL1 (Figure 7). Based on the findings, we exhibited a novel mechanism of radiotherapy to augment CD8^+^ T cells immunity.

## Figures and Tables

**Table 1:** The primer sequence for qPCR.

Gene	Direction	Primer Sequence
*GAPDH*	Forward	GAGTCAACGGATTTGGTCGT
	Reverse	TTGATTTTGGAGGGATCTCG
*GZMB*	Forward	ACTGCAGCTGGAGAGAAAGG
	Reverse	TTCGCACTTTCGATCTTCCT
*CD274 (PD-L1)*	Forward	GTACCTTGGCTTTGCCACAT
	Reverse	CCAACACCACAAGGAGGAGT
*TNFRSF14 (HVEM)*	Forward	CCACTGGGTATGGTGGTTTC
	Reverse	TCACCTTCTGCCTCCTGTCT
*LGALS9 (Galectin-9)*	Forward	CCTTTGACCTCTGCTTCCTG
	Reverse	AAACAGACAGGCTGGGAGAA
*STAT1*	Forward	CCGTTTTCATGACCTCCTGT
	Reverse	TGAATATTCCCCGACTGAGC
*STAT2*	Forward	GAGGCCTCAACTCAGACCAG
	Reverse	GCGTCCATCATTCCAGAGAT
*STAT3*	Forward	TTTCACTTGGGTGGAGAAGG
	Reverse	GCTACCTGGGTCAGCTTCAG
*PSM88*	Forward	CACGGGTAGTGGGAACACTT
	Reverse	TCACCCAACCATCTTCCTTC
*PSMB10*	Forward	AATGTGGACGCATGTGTGAT
	Reverse	TCCAGGGTTAGTGGCTTCAC
*NFKB2*	Forward	GAACAGCCTTGCATCTAGCC
	Reverse	TCCGAGTCGCTATCAGAGGT
*SOCS3*	Forward	GCCACCTACTGAACCCTCCT
	Reverse	ACGGTCTTCCGACAGAGATG
*UBE2L6*	Forward	CAACCTCCCTACCACCTGAA
	Reverse	GCAAGGCTTCCAGTTCTCAC
*EFNA1*	Forward	GGTGACTGTCAGTGGCAAAA
	Reverse	AGTGGAAGGAGCAGCACAGT
*PML*	Forward	ACACAACGTGAGCTTCATGG
	Reverse	AAGTGGGGTGGAGACTCCTT
*FOSL1*	Forward	AGCTGCAGAAGCAGAAGGAG
	Reverse	GGAGTTAGGGAGGGTGTGGT
*MCL1*	Forward	AGAAAGCTGCATCGAACCAT
	Reverse	CCAGCTCCTACTCCAGCAAC
*ISG15*	Forward	TGTCGGTGTCAGAGCTGAAG
	Reverse	GCCCTTGTTATTCCTCACCA
ChIP_STAT1/3_*MCL1*	Forward	AAAAGGGCTCACAAATCAGGT
	Reverse	GTCTTCGGAGGCTCTGAGTG
ChIP_STAT1/3_*PD-L1*	Forward	ACTAGCATGGCTGAGACAGTGA
	Reverse	CATACCTAGTAGAACCTGCCCTGT

**Table 2:** The target sequence of shRNA plasmids.

Gene	Clone ID	Targeted Sequence
*Luciferase*	TRCN0000072249	GCGGTTGCCAAGAGGTTCCAT
*STAT1*	TRCN0000004266	CGACAGTATGATGAACACAGT
*STAT3*	TRCN0000020842	CACAATCTACGAAGAATCAA.
*MCL1*	TRCN0000005515	GCAGAAAGTATCACAGACGTT
*PDL1*	TRCN0000056914	CGAATTACTGTGAAAGTCAAT

## Supplementary Materials

The following are available online at https://www.mdpi.com/article/10.3390/cells10102515/s1, Table S1: Gene profiling expression of A549 treated with IFNγ using RNAseq.
